# Dr. Ernest Alfred Snape

**Published:** 1909-05-22

**Authors:** 


Dr. Ernest Alfred Snape, honorary physician to the Cripples' Home, St. Marylebone, and the Governesses' Benevolent Institution, died last week in London from appendicitis. He was educated at Charing Cross Hospital, University College, London, and the University of Brussels, where he took the M.D. degree in 1893, after qualifying in London six years previously. He was captain in the

R.A.M.C. Territorial Force and Past-President of the Brussels Medical Graduates' Association.

